# Key performance and training parameters in primary total hip arthroplasty – an expert consensus using the Delphi technique

**DOI:** 10.1177/11207000211056864

**Published:** 2021-11-08

**Authors:** Daniel Howgate, Patrick Garfjeld Roberts, Ben Kendrick, Jonathan Rees

**Affiliations:** 1 University of Oxford Nuffield Department of Orthopaedics Rheumatology and Musculoskeletal Sciences, The Botnar Research Centre, Oxford, UK; 2 NIHR Oxford Biomedical Research Centre, The Joint Research Office, Oxford, UK; 3 Oxford University Hospitals NHS Foundation Trust, Nuffield Orthopaedic Centre, Oxford, UK; 4 Dinwoodie Charitable Company, Crawley, UK

**Keywords:** Delphi, expert consensus, medical education, surgical training, total hip arthroplasty, total hip replacement

## Abstract

**Aims:**

Primary total hip arthroplasty (THA) is a commonly performed and successful operation which orthopaedic trainees must demonstrate competence in prior to completion of surgical training. An assessment of agreement between surgical trainers regarding the critical steps of a primary THA has never been undertaken. The aim of this study was to define and rank the key steps of a primary THA regards ease of teaching and their importance in achieving the best patient outcome.

**Materials and methods:**

The Delphi technique with 3 iterative rounds was used to establish expert group consensus. The benchmark for consensus was set at an 80% agreement in any category for each step of a THR. The intra-class correlation coefficient (ICC) was used to report on the inter- and intra-rater reliabilities between and within participants responses respectively in rounds 2 and 3.

**Results:**

50 consultant orthopaedic hip surgeons completed round 2, and 28 completed round 3. Overall, 27 steps (54 parameters) were identified, with 16 parameters achieving consensus agreement for their impact on patient outcome, and 17 for ease of teaching. The inter-rater ICC for patient outcome parameters was 0.89 and 0.92 in rounds 2 and 3 respectively while for teaching parameters it was 0.82 and 0.73. 50% of surgeons agreed that acetabular reaming, assessing and accurately restoring leg length, and acetabular cup anteversion were the 3 most difficult steps to teach trainees, while 90% agreed these 3 steps were substantially important to patient outcome. Another 5 steps achieved consensus for their substantial impact on patient outcome but failed to achieve consensus for ease of teaching.

**Conclusions:**

The results of this expert consensus have produced a rank-order list of the key steps in primary THA, which may be used for orthopaedic curriculum development and guiding focused improvements for surgical training in primary THR including simulation.

## Introduction

Primary total hip arthroplasty (THA) is the most commonly performed and successful orthopaedic operation of the 21st Century.^1–3^ The process of training orthopaedic surgeons to competently perform operations such as a primary THA requires the accumulation of scientific knowledge, technical skills, operative experience, professional judgement, alongside sufficient supervision until competence is achieved. This process is time-consuming and susceptible to variations in the healthcare systems and environment in which surgeons are now trained. Restrictions in both working times, and the volume and breadth of in-training surgical experience within contemporary surgical training programmes therefore require an increasingly focussed approach in order to maximise the opportunities for trainees to achieve operative competence prior to completion of their training.^4,5^

Despite its prevalence and importance, there are no agreed teaching methods for THA overall, or for any of its key stages. Furthermore, it is unknown if surgeons even agree or disagree about the key stages and how best to teach them. In order to develop focused and effective surgical training it is critical to firstly define the key steps of the procedure. Defining and categorising these key steps in order of importance can be a challenge in itself as there is a natural variation across surgeons who perform the same operation. Besides the surgeon’s technical ability to perform each of these steps, it is also important to consider their ability to teach each step. An individual step may be judged by a trainer both according to how critical it is to patient outcome, and how easy it is to teach. Obtaining a balanced perspective from a large cross section of experienced surgeons who are actively involved in contemporary training offers the opportunity to improve surgical training in THA. Such consensus group methodologies are defined as a systematic means to assess, develop, and define levels of agreement between individuals.^6^ The Delphi technique is now a recognised form of consensus methodology used in formulating an opinion within a group of experts in their domain of expertise.^7^

The aim of this study was to define the key steps of a primary THA, rank them in order of importance for patient outcome, and on ease of teaching to surgical trainees. The results generated will inform orthopaedic trainers, trainees and training standard committees about the areas of THA training viewed as either critical to patient outcome or difficult to teach and that would benefit from particular focus or further strategies to enhance training.

## Methods

### Study and questionnaire design

Considerable variability and quality have been demonstrated in the application, structure, execution, and reporting of consensus methodologies in the healthcare setting.^8,9^ In this study a pre-determined maximum of 3 rounds was set for achieving group consensus, including the literature review and pilot survey.

The first round generated a list of key steps of a primary THA using information from published orthopaedic journals and textbooks, and canvassing opinion from a group of expert hip surgeons at a specialist orthopaedic hospital using a pilot survey. This survey contained a provisional list of discrete steps involved in primary THA, open-ended questions, and space for free-text responses in order to gather and refine the final list of steps. The steps identified were sub-divided into outcome and training parameters in order to reflect the impact of each step on the outcome of the operation, and whether surgeons find it easy to teach the step to surgical trainees. The outcome parameter was assessed using a 3-point Likert scale, and the training parameter was assessed using a binary ‘yes/no’ answer. These scales were specifically selected to reduce any ambiguity in response and remove any requirement for ‘grouping’ responses which may be caused by increasing the number of response categories.^10^

In round 2, questionnaires were circulated to attendees throughout the 2019 British Hip Society Annual Scientific Meeting. This meeting was selected for distribution of the questionnaire as it is the national conference for orthopaedic surgeons with a sub-specialty interest in hip surgery and therefore attracts a high concentration of eligible participants. The eligibility criteria for participation in this study were clearly defined on the questionnaire as any consultant orthopaedic hip surgeon with an active or recent (within 5 years) role in surgical training. All completed questionnaires from surgeons not meeting the eligibility criteria were removed prior to analysis. Information on participant demographics and clinical profiles were collected. Following an interval of 3 months, a third round was conducted remotely via e-mail to all round 2 participants. All steps that achieved group consensus in round 2 were removed prior to distributing the revised questionnaire in round 3. The electronic questionnaires used in round 3 were constructed using a template on the online platform google forms (Google Forms; Google LLC, Mountain View, CA, USA). A series of 3 reminder notifications were sent at fortnightly intervals to all participants who did not complete the questionnaires. Responses from round 3 were closed at 8 weeks following the initial contact, and all participants who had not responded by this time point were excluded from further involvement, however their responses from round 2 were included in the data analysis.

### Data analysis

A unified definition of consensus when performing a Delphi analysis does not exist in the literature.^11^ The benchmark for ‘group consensus’ in this study was set high at 80% agreement in any 1 category on the 3-point Likert or binary scale for each THA step, which is in keeping with the recommendations of many authors.^12,13^ The inter-rater reliability (IRR) in rounds 2 and 3 are reported using a 2-way random effect, consistency, average-measures intra-class correlation co-efficient (ICC) with a 95% confidence interval (CI) to assess the reliability of participants ratings of the parameters for each step.^14,15^ The intra-rater stability of responses between round 2 and 3 for all parameters which do not meet group consensus are reported using a 2-way mixed effect, absolute agreement, single-measures ICC with a 95% CI. Statistical analysis and graphical visualisation were performed using RStudio version 1.2 (RStudio Team (2020): Integrated Development for R. RStudio, PBC, Boston, MA, USA).

## Results

### Round 1

A list of 26 discrete steps in performing a primary THA was generated using the findings from the literature review and pilot survey. These were used to construct a data capture questionnaire for distribution to eligible participants in round 2.

### Round 2

50 consultant hip surgeons provided complete responses to the questionnaire in round 2. Of these participants, 14 reported <5 years of experience as a consultant, 13 had between 5–10 years’ experience, 5 had between 10–15 years’ experience, 11 had between 15–20 years’ experience, and 7 >20 years’ experience. Overall, 17 participants worked within a university teaching hospital, and 33 worked within district general hospitals. 48 were actively involved in training orthopaedic specialty registrars or fellows, and all participants had trained surgeons within the last 5 years. 42 were currently clinical supervisors of specialty registrars or fellows (Table 1).

**Table 1.** Round 2 participant clinical profiles.

	Respondents (%)
Consultant experience	
<5 years	14 (28%)
5–10 years	13 (26%)
10–15 years	5 (10%)
15–20 years	11 (22%)
>20 years	7 (14%)
Hospital setting	
University teaching	17 (34%)
District general	33 (66%)
Trainer status	
Clinical supervisor	42 (84%)
Actively training	48 (96%)
Trainer in the last 5 years	50 (100%)

Digital preoperative templating was routinely used by 41 participants (82%), in comparison to 6 who manually templated (12%), and 3 who did not routinely template (6%). A lateral decubitus patient position was used by 46 participants (92%), with 4 reporting the use of a supine position (8%). A posterior surgical approach was routinely used by 43 participants (86%), with 6 reporting the use of a Hardinge (direct lateral) approach (12%), and 1 using a direct anterior approach (2%). Hybrid implant fixation (uncemented acetabular cup with cemented femoral stem) was preferred by 23 participants (46%), followed by all cemented implants by 15 participants (30%), all uncemented implants by 5 participants (10%), a mixture of implant fixation modes by 4 participants (8%), and reverse hybrid fixation by 3 participants (6%) (Table 2).

**Table 2.** Round 2 participant surgical preferences.

	Respondents (%)
Preoperative templating	
Digital	41 (82%)
Manual	6 (12%)
None	3 (6%)
Patient positioning	
Lateral	46 (92%)
Supine	4 (8%)
Surgical approach	
Posterior	43 (86%)
Hardinge	6 (12%)
DAA	1 (2%)
Implant fixation	
Hybrid	23 (46%)
Cemented	15 (30%)
Uncemented	5 (10%)
Reverse hybrid	3 (6%)
Mix	4 (8%)

Data on the operative profiles for primary and revision THA of each participant were obtained from the publicly available section of the United Kingdom’s National Joint Registry (NJR) website (https://surgeonprofile.njrcentre.org.uk/). Data were not available for a total of 5 participants. Of the remaining 45 participants, the mean number of primary and revision THA performed was 372 and 51 over 36 months.

### Round 2 responses

**Figure 1.**
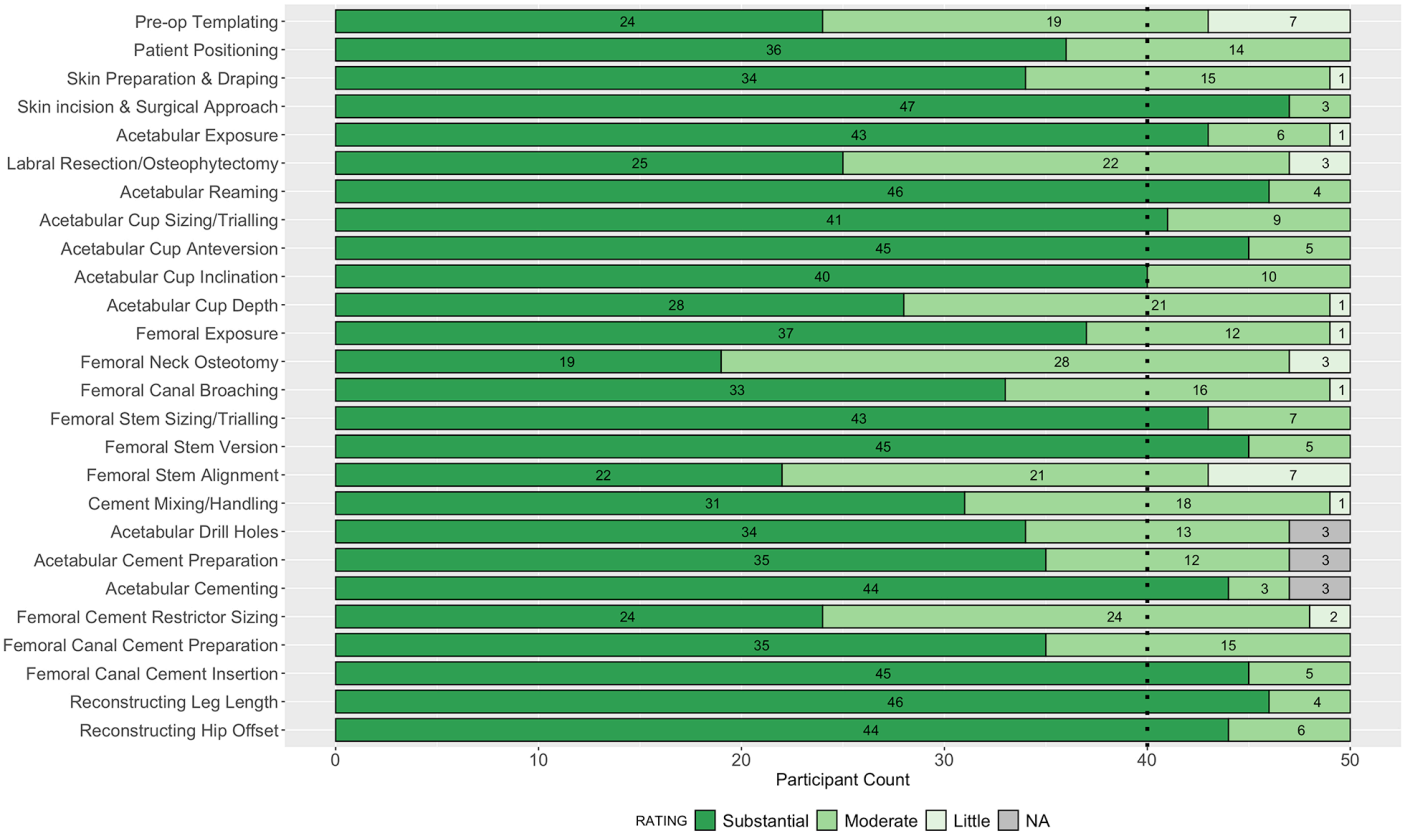
Stacked bar plot of participant ratings in round 2 for the impact of each step on the outcome of the operation. Dashed line indicates 80% consensus threshold.

**Figure 2.**
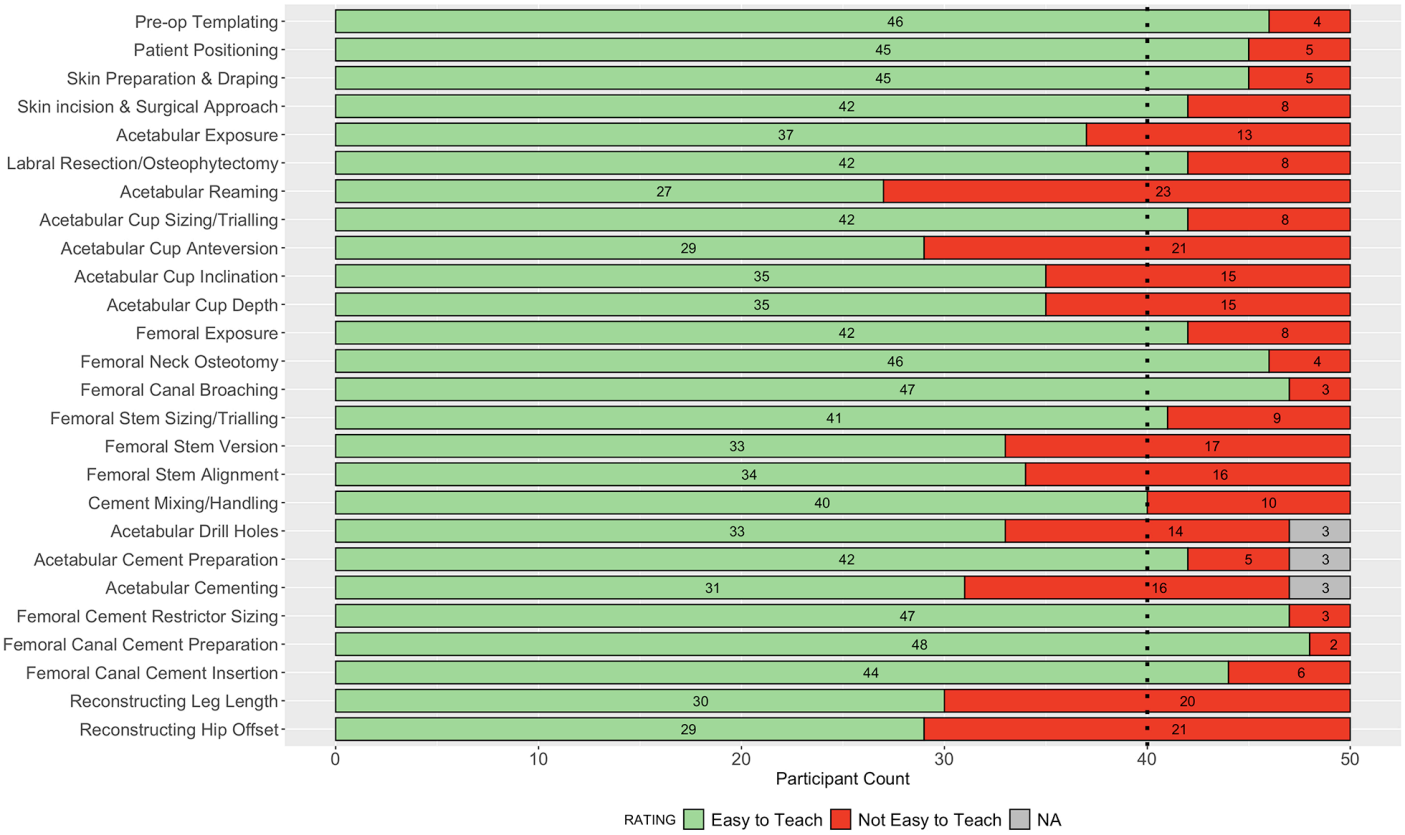
Stacked bar plot of participant ratings in round 2 for the ease of teaching each step of the operation. Dashed line indicates 80% consensus threshold.

Consensus agreement was achieved in 27 of 52 parameters assessed: 12 for importance on patient outcome, and 15 for ease of teaching. The following steps achieved >90% consensus for their substantial impact on patient outcome following primary THA: skin incision and surgical approach; acetabular reaming; reconstructing leg length; acetabular cup version; femoral stem version; and femoral cement insertion. Overall, ⩾40% of participants rated the following steps as not easy to teach to surgical trainees: acetabular reaming; acetabular cup version; reconstructing hip offset; and leg length. The collective responses from all 50 participants in round 2 are summarised in Figures 1 and 2.

The inter-rater reliability across all participants was 0.89 (CI, 0.8–0.94) for the impact on outcome parameters, and 0.82 (CI, 0.73–0.89) for the ease of teaching parameters. A total of 3 participants recorded non-applicable responses to the steps on acetabular cementing, as they did not use this technique in their practice. Following review of the free text responses, 1 further step (soft tissue handling and wound closure) was added for assessment in round 3.

### Round 3 responses

**Figure 3.**
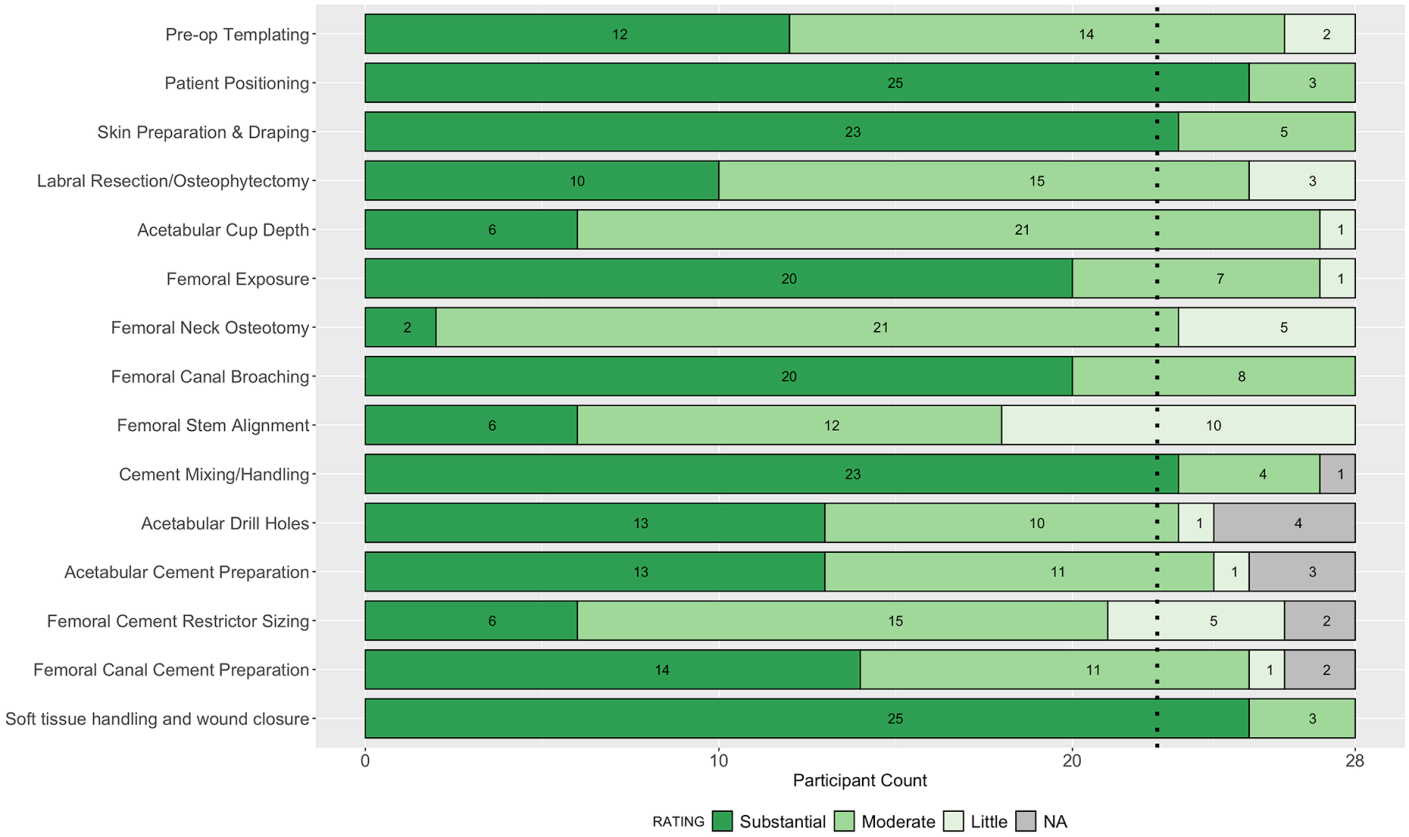
Stacked bar plot of participant ratings in round 3 for the impact of each step on the outcome of the operation. Dashed line indicates 80% consensus threshold.

**Figure 4.**
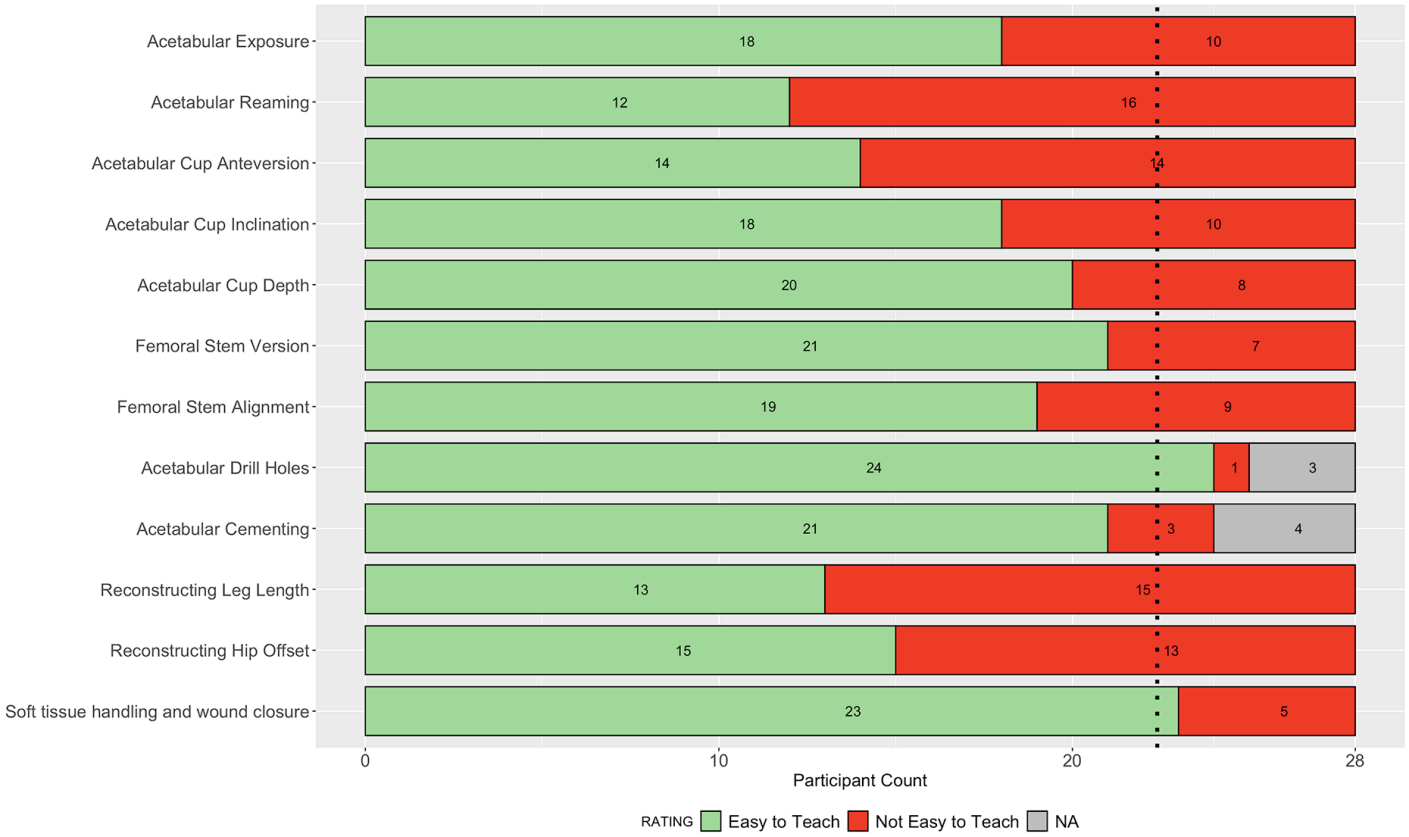
Stacked bar plot of participant ratings in round 3 for the ease of teaching each step of the operation. Dashed line indicates 80% consensus threshold.

A total of 27 parameters were assessed in the questionnaire in round 3: 15 for importance on outcome, and 12 for ease of teaching. 25 parameters were from round 2, where consensus had not been achieved, and a further 2 from the additional step of soft tissue handling and wound closure. Complete responses were received from 28 participants in round 3, and consensus agreement was achieved in a further 6 parameters. The 4 parameters achieving consensus for importance on outcome were: patient positioning, soft tissue handling and wound closure, skin preparation and draping, and cement mixing and handling. The 2 parameters achieving consensus for ease of teaching were: acetabular drill holes, and soft tissue handling and wound closure. The collective responses in round 3 are summarised in Figures 3 and 4.

The inter-rater reliability across all participants was 0.92 (CI, 0.86–0.96) for the impact on outcome parameters, and 0.73 (CI, 0.73–0.89) for the ease of teaching parameters. Following round 3, a total of 21 parameters remained without group consensus: 11 for impact on patient outcome, and 10 for ease of teaching.

### Change in response between round 2 and 3 for parameters not achieving consensus

Between rounds 2 and 3 participant responses changed <25% for 3 parameters; <50% for 16 parameters; and >50% for 2 parameters. The 3 parameters for which participants responses changed <25% were: the impact of preoperative templating on patient outcome, and the ease of teaching both acetabular cup inclination and acetabular reaming. The 2 parameters for which participants responses changed >50% were: the impact of femoral cement restrictor sizing, and femoral stem alignment on patient outcome.

**Figure 5.**
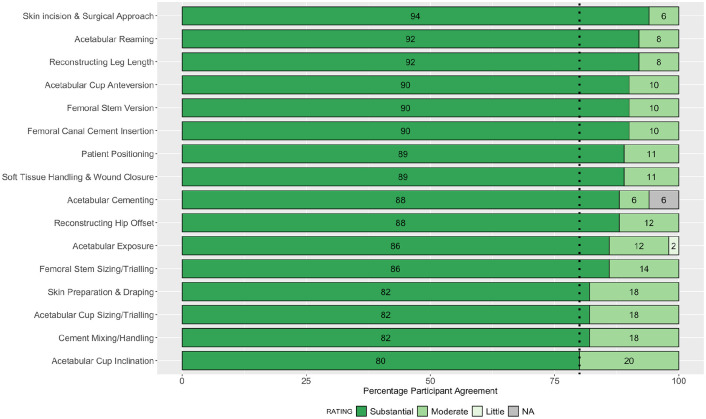
Stacked bar plot showing the rank-order of steps achieving consensus for importance on the outcome of the operation. Dashed line indicates 80% consensus threshold.

**Figure 6.**
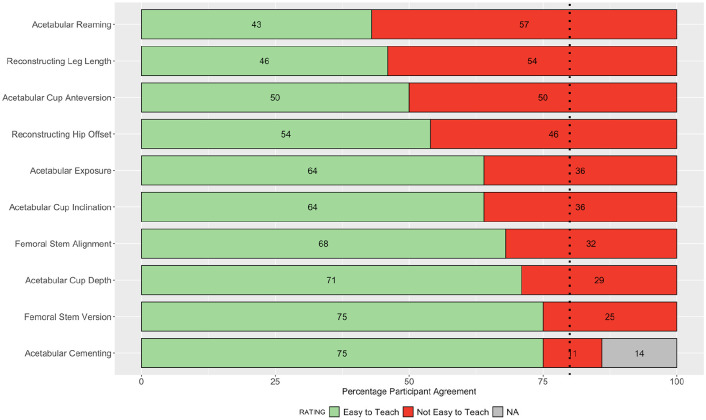
Stacked bar plot showing the rank-order of steps not achieving consensus for ease of teaching surgical trainees. Dashed line indicates 80% consensus threshold.

Overall, 16 parameters achieved an 80% consensus agreement for their impact on the outcome of the operation, and 17 for ease of teaching. The rank order of parameters achieving consensus for importance on outcome of the operation, alongside the parameters which did not achieve consensus for ease of teaching (therefore perceived as difficult to teach) were of primary interest (Figures 5 and 6).

A total of 8 steps reached consensus for both importance on their impact on the outcome of the operation and did not achieve consensus for ease of teaching (Table 3).

**Table 3.** Steps achieving consensus agreement for their impact on the outcome of the operation, and not achieving consensus for their ease of teaching.

Step	% Rated as having a substantial impact on the outcome of the operation (rank order)	% Rated not easy to teach (rank order)
Acetabular reaming	92% (2)	57% (1)
Assessing & accurately reconstructing leg length	92% (2)	54% (2)
Acetabular cup anteversion	90% (3)	50% (3)
Assessing & accurately reconstructing hip offset	88% (5)	46% (4)
Acetabular exposure	86% (6)	36% (5)
Acetabular cup inclination	80% (8)	36% (5)
Femoral stem version	90% (3)	25% (9)
Acetabular cement insertion & pressurisation	88% (5)	11% (10)

## Discussion

Several steps identified within this study are recognised as having a substantial impact on patient outcome following primary THA, most notably: preoperative templating,^16^ patient positioning,^17^ surgical approach,^18^ acetabular cup and femoral stem positioning,^19–21^ restoration of femoral offset,^22,23^ balancing leg lengths,^21^ and wound closure.^24^ What this study adds to the scientific literature is an agreed rank-order list of the key steps involved in primary THA to achieve the best patient outcomes, as well as defining the steps that are difficult to teach surgical trainees. Differences between surgical trainees and experienced orthopaedic surgeons have been noted in relation to operative timings, patient complications, revision rates, and outcomes following primary THA.^25–28^ However, specific areas for improvement in training in order to overcome these differences have not been identified or recommended. There is a need to develop novel methods for improving training and performance in primary THA, and the results of this expert consensus form the first step in this process.

Participant identification and selection are critical components of the Delphi technique, as the quality of participants dictates the results generated. No specific criteria exist to define an expert in hip surgery however, experts should generally be well-regarded representatives of their profession; highly trained, knowledgeable, and competent within the specialised area related to the target issue; and have the ability to implement the findings of the study.^7^ The strengths of this study include attention to the clinical profiles of the expert participants, notably the hospital setting, years of experience, and operative numbers which are representative of the wider orthopaedic community. The ratings of these participants therefore provide a good insight into and reflection of contemporary surgical practice and training. According to NJR figures, the mean 36-month primary and revision THA procedures performed by participants within our study was considerably higher than the national average, indicating that these participants are representative of a high-volume group of surgeons with the concomitant increase in experience and may therefore be justly regarded as experts. Using this relatively large demographic of expert participants in combination with adherence to the pre-determined and robust Delphi technique methodological criteria and using a high threshold for defining consensus adds validity to our findings.

While this study identifies consensus for 33 out of 54 parameters, a total of 21 parameters remained without group consensus. The 10 parameters that did not achieve consensus for ease of teaching are of particular interest as they constitute the steps which surgeons perceive as difficult to teach trainees (Figure 6). A simulation-based approach to teaching these steps to trainees in a non-clinical environment may therefore be a safer and more effective training modality prior to refinement of these skills in clinical practice. All 16 parameters achieving consensus for impact on the outcome of the operation were rated in the substantial category on a 3-point Likert scale (Figure 5). It is important to highlight that the 11 parameters which did not achieve consensus for impact on the outcome of the operation remain important steps of a THA. The fact that consensus was not achieved may be a reflection of our high threshold, or that participants ratings on the degree of their individual impact (substantial/moderate/little) were more evenly distributed for these steps. A total of 9 out of 11 parameters which did not achieve consensus for impact on the outcome of the operation had kappa values <0.5, indicating poor intra-rater reliability. This reflects the change in participants ratings for these parameters between rounds of the study. Conducting further iterative rounds of the Delphi process may theoretically increase intra-rater reliability, however, we believe that the risk of participant fatigue, increased drop-out rates, and diminishing returns outweighed this potential benefit.

A limitation of the Delphi technique is that it does not allow participants to discuss, debate, or elaborate their views on the issues raised within the questionnaire. However, the free-text responses within round 2 allowed participants to raise their ideas regarding amendments or further steps to be added within the questionnaire, which in this study resulted in an additional step being included in round 3. Another potential weakness relates to the preferred implant fixation used by surgeons. Comparison of the participants’ implant fixation preferences to the NJR data suggest that users of hybrid fixation were over-represented, and so users of all uncemented implants were potentially under-represented within this study. A participant’s choice of implant fixation may affect their individual attitudes and subsequently their ratings of the importance of specific steps of this operation. For example, surgeons who routinely use uncemented femoral stems may place a greater emphasis on the femoral neck osteotomy as this largely dictates leg length on the femoral side. Furthermore, 3 surgeons did not respond to the steps involving cemented acetabular cups as this was not applicable to their practice. Such limitations are a consequence of using a pragmatic questionnaire which was aimed at capturing and assessing the breadth of orthopaedic practice and implant combinations.

The results of this study will be particularly useful in guiding focussed interventions for optimising orthopaedic surgical training in primary THA, ultimately aimed at reducing complications and adverse patient outcomes. Consequently, we have already developed a simulation-based course for orthopaedic trainees at our institution, which focusses on the highly ranked critical steps of a primary THA. This course delivers the core scientific knowledge and theory underpinning a successful THA, allows trainees the opportunity to practise key steps of this operation in the safety of a simulated environment, and uses validated methodology to assess trainees’ attainment of practical surgical skills in the critical steps of this operation (e.g., acetabular and femoral preparation, cementing technique, and implant positioning). The impact of this focussed and evidence-based course on knowledge and operative performance (both in a simulated and theatre environment) is currently being assessed in a registered clinical randomised-controlled trial (clinicaltrials.gov ID NCT04267172). Orthopaedic surgeons who are actively involved in teaching and training along with their trainees can use the findings of this study as a framework to identify specific areas for personal development. Similarly, local hospitals, regional networks, and even national organisations responsible for developing curricula and delivering post-graduate medical education may decide to enhance both simulation-based and clinical training opportunities in the steps identified as critical to the outcome or perceived as difficult to teach ‘on the job’.

## Conclusion

This expert consensus on the key performance and training parameters in primary total hip replacement has produced a rank-order list of steps which may be used by organisations, trainers and trainees in both curriculum development and enhancing surgical training opportunities specific to the aspects of this operation and viewed as important to patient outcomes and/or difficult to teach. This may help to focus and streamline surgical training, optimise surgeon performance, and further improve outcomes for future THA patients.
